# Dietary Changes, Motivators, and Barriers Affecting Diet and Physical Activity among Overweight and Obese: A Mixed Methods Approach

**DOI:** 10.3390/ijerph182010582

**Published:** 2021-10-09

**Authors:** Merete Hagen Helland, Gerd Lise Nordbotten

**Affiliations:** Department of Education and Sports Science, University of Stavanger, 4021 Stavanger, Norway; gerd.l.nordbotten@uis.no

**Keywords:** obesity, overweight, intervention, motivators, barriers, diet, exercise

## Abstract

The aims of this study were to examine (1) effects of nutritional guidance and physical activity on dietary habits among overweight and obese after an intervention and one year after follow-up (quantitative study) and (2) barriers and motivators for changes in diet and physical activity (qualitative study). A total of 98 participants with a mean age of 46.8 ± 10.2 years were included and divided into a Training Group (TG) (n = 51) or a Nutritional Guidance and Training Group (NTG) (n = 47). At baseline, after 33 weeks, and one year after the intervention, participants answered a questionnaire. Interviews gave data to elicit motivations and challenges related to diet and exercise. A GLM repeated measures analysis was used to investigate differences and interactions between factors. Participants ate healthier after starting to exercise. After 33 weeks, the NTG ate significantly more vegetables (*p* = 0.026) and legumes (*p* < 0.01) than the TG. No significant differences were found one year after follow-up. General health was the most important motivator for changing diet and exercise. Barriers to changing diet were related to work, family, meal size, and participants’ internal decisions to change habits. Barriers to exercise were holidays and time constraints. Planning purchases and regular exercise were important factors to achieve and maintain weight loss.

## 1. Introduction

A well-balanced diet and physical activity are important for good health, but a combination of increased activity and a healthier diet is probably the best way to maintain a stable, lower weight in the long run [1,2,3,4] and avoid future health issues [5]. Losing weight might also be easier than maintaining a stable, lower weight. Weight compensation due to increased food intake is common after starting up aerobic exercise and may explain the low weight loss in intervention studies [6]. The Stages of Change Model describes when and how people change non-desirable behaviors, with change defined as a dynamic variable with five to six discrete stages: (1) precontemplation (not yet acknowledging that there is a problem behavior that needs to be changed, (2) contemplation (acknowledging that there is a problem, but not ready to make a change), (3) determination (getting ready to change), (4) action (change willpower), and (5) maintenance (maintaining the behavior changes) [7,8]. A meta-analysis of non-surgical weight loss interventions in obese adults found that 28.4% of participants dropped out of the study prior to the maintenance phase [9]. Some will also experience relapse and a return to older behavior and abandon all new changes [7,8]. In a study by Meffert and Gerdes [10], almost half of those who dropped out during the study reported job demand (49.2%) or family obligations (40.5%) as reasons for quitting participation in the program.

To successfully achieve or maintain weight loss, Fuglestad et al. [11] and Neve et al. [12] stated the importance of not using food to control emotions and mood, together with regular meals (not skipping meals), avoiding having snacks in the house, and eating take-away meals less frequently.

The most important motivation factors related to changes in diet, according to Satia et al. [13], were self-image and personal health. However, both demographic characteristics and baseline diet influenced motivation, and self-image was more important among women, just as personal health was more important among older men [13]. According to Aaltonen et al. [14], the most frequently reported motives for being physically active were physical fitness, health maintenance, and psychological well-being. Higher adherence has also been linked to social support, older age, higher income, and education, although support from children showed no effect [15]. Lower adherence has been linked to low age, higher weight, low socioeconomic status, past negative experiences with physical activity, depressed mood, and more children living at home [16,17,18,19].

Christofaro et al. [20] showed that physical activity is positively associated with healthier eating habits. Additionally, Perea Sánchez et al. [21] found that active, young adults follow a healthier diet than sedentary young adults. However, more documentation of how and when these changes occur is needed, to which this study contributes. Evidence of what optimizes the effectiveness of group-based lifestyle interventions remains limited and more evidence is needed [13,22].

The objectives of the present study were to examine (1) effects of nutritional guidance and physical activity on dietary habits among the overweight and obese after an intervention and at one year after follow-up and (2) barriers and motivators for changes in diet and physical activity.

## 2. Materials and Methods

The study was an analytical, observational case-control study organized as a randomized controlled trial. A mixed methods design with an explanatory sequential design structure was used, whereby the quantitative phase was followed by a qualitative phase, to elaborate the quantitative results [23]. The qualitative analysis was based on an inductive, thematic analysis [24].

A total number of 98 participants (65 women), with a mean age of 46.8 ± 10.2 years, were included in this project. More women than men participated, but the completion rate and reasons for participating in the project were approximately equal. Women and men had a dropout rate of 40% and 42%, respectively. The study was approved by the Regional Committees for Medical and Health Research Ethics (REK) in Norway (project number 2016/1099). All participants provided signed written informed consent to take part in the study.

**Figure 1:**
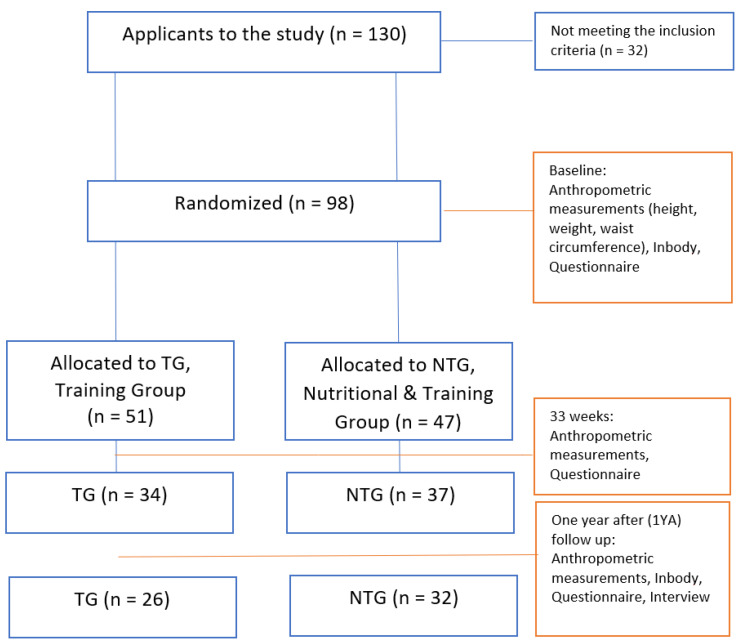
Flow charts of participants and measurements through the study. Details regarding anthropometric measurements and running time can be found in Helland et al. [26], Nordbotten et al. [27], and Saxeide [28].

Participants were recruited among applications from the readers of a local newspaper (general invitation to the public in Stavanger Aftenblad) after fulfilling the inclusion criteria: BMI ≥ 25, no medical conditions, no problems walking, no pain or discomfort, and a desire to become fitter and reduce their BMI. A prerequisite for participating in the project was that the participants were able to participate weekly in two joint trainings and two individual training sessions. Body mass index (BMI) is used to define overweight and obesity; a BMI higher than 25 kg/m^2^ is used as an index of overweight [25]. Exclusion criteria were BMI ≤ 25, minors, metabolic diseases, pain or discomfort, problems walking, and a previous psychological history or eating disorder. The study was carried out in Rogaland County in Norway from 2016 until 2018. A flow chart of participants is shown in Figure 1.

Research Randomizer (Research Randomizer Version 4.0; www.randomizer.org, accessed on 1 October 2016) was used to allocate participants into two groups. Research Randomizer is a free resource to generate random numbers or assign participants to experimental conditions. Participants in the same household (married/cohabitants) were randomized as one, to minimize the external influence of each other’s diet. One group received nutritional guidance (Nutritional Training Group = NTG); and the other group was instructed to eat as they did before entering the project (TG = Training Group). All participants, independent of group, followed a training program with two weekly, supervised training sessions at 4.30 p.m. Monday and Thursday, consisting of 10 min of warm up, 30 min of high-intensive interval running/walking depending on physical capacity, and 10 min of general strength training. In addition, participants were to take two weekly alternative training sessions with a moderate intensity of minimum 30 min each. Activity and duration were reported in training diaries and followed up regularly throughout the intervention period. In addition to training, the NTG also received nutritional guidance (10 × 2 h) and practical cooking lessons (8 × 3 h). To minimize absence, the cooking lessons were duplicated. Participants did not follow any defined, low-calorie diet, but received general nutritional guidance regarding which food to buy and the size and composition of meals to reduce their daily calorie intake. Participants were advised to follow 11 dietary guidelines published by the Norwegian Directorate of Health [29]: (1) enjoy a varied diet with lots of vegetables, fruit and berries, wholegrain foods, and fish, as well as limited amounts of processed meat, red meat, salt, and sugar; (2) maintain a good balance between the amount of energy obtained through food and drink and the amount of energy expended through physical activity; (3) eat at least five portions of vegetables, fruit, and berries every day; (4) eat wholegrain foods every day; (5) eat fish two to three times a week, also as a spread on bread; (6) choose lean meat and lean meat products, and limit the amount of processed meat and red meat; (7) include low-fat dairy foods in your daily diet; (8) choose edible oils, liquid margarine, and soft margarine spreads instead of hard margarines and butter; (9) choose foods that are low in salt and limit the use of salt when preparing food and at the table; (10) avoid food and drink that are high in sugar; and (11) choose water as a thirst quencher. In addition to these 11 guidelines related to diet, there is also one guideline on recommendations for physical activity. These guidelines are recommended for all healthy individuals, independent of size and weight. TG participants were requested to maintain their normal diet during the 33-week intervention period and were offered nutritional lessons and practical cooking lessons after this period.

SurveyXact (Rambøll Management Consulting, Aarhus, Denmark) was used as an online survey platform to develop a questionnaire to gather information regarding participants’ reasons for joining the project, as well as thoughts and attitudes concerning food/diet and exercise. The questionnaire consisted of five sections. The first included demographic variables (gender, level of education, occupation, age, country of residence, and household size). Dependent variables were divided into questions regarding food frequency, strength training (frequency, duration, type), endurance training (frequency, duration, type), and how to cope with daily life. This article focuses on diet and questions related to barriers and motivators affecting physical activity and diet. The questionnaire was conducted electronically at project start, after the intervention period (33 w), and one year after the intervention period.

A food frequency questionnaire (FFQ) approach was used to provide estimates of usual dietary intake over time. The FFQ lists specific foods and asks the participant if they eat them and if so, how often and how much they eat, being dependent on participants’ own dietary recall. Questions used were adapted from a validated FFQ [30]. Participants were asked to report their usual intake, expressed as frequency (0–1 servings/month, 2–3 servings/month, 1–3 servings/week, 4–6 servings/week, and 1–2 servings/day). Frequencies of consumption were converted during data analysis to servings per week (0–1 times/month = 0.5/7 = 0.125 servings/week, 2–3 servings/month = 0.625 servings/week, 1–3 servings/week = 2 servings/week, 4–6 servings per week = 5 servings/week, and 1–2 servings/day = 10.5 servings/week). Healthy foods assessed were fruit, vegetables, legumes, fish, and wholegrains. Unhealthy foods were sugar-sweetened beverages (SSB), junk food (pizza, hamburgers, french fries, etc.), sweets (candy/chocolate), snacks (salty snacks such as potato crisps), refined grains, and sweet pastries (cakes, cookies, etc.). Questions regarding reasons to have a healthy diet and an active lifestyle were adapted from the Treatment Self-Regulation Questionnaire [31]. All participants were asked about reasons for (or not) participating in joint training, changing their diet, and reasons for not achieving the expected weight loss. In addition, a total of four randomly chosen participants, two from TG and two from NTG, were also interviewed during and after the project period, to elaborate and elicit their motivations and challenges related to diet and exercise. A general linear model (GLM) repeated measures analysis was conducted to evaluate whether there was a difference in food intake over time after participation in the intervention study to detect differences between groups and interaction effects. All measurements were adjusted for multiple comparisons with the Bonferroni procedure. All statistical analyses were performed using IBM SPSS Statistics Version 25.0 (IBM, Amonk, NY, USA). Preliminary analyses were performed to ensure that assumptions concerning normality, linearity, and homoscedasticity were not violated. Values for skewness and kurtosis were at ± 2, indicating a normal univariate distribution for all parametric data. The alpha level for significance was set at *p* < 0.05. Data are presented as a mean (M) ± the standard deviation of the mean (SD). For differences, the 5th95th percentiles are presented.

Method triangulation was used to study motivation and barriers related to exercise and diet as both questionnaire and interview were used. All participants answered the questionnaire and to elucidate these answers, four participants (two from each group) were randomly chosen to participate in the interviews. A five-step process was used to develop a semi-structured interview guide [32]: (1) identifying the prerequisites for using semi-structured interviews; (2) retrieving and using previous knowledge; (3) formulating the preliminary semi-structured interview guide; (4) pilot testing the guide on two persons not related to the project, in order to measure its reliability and validity prior to the main interviews; and (5) presenting the complete semi-structured interview guide. The interview guide was divided into two themes—(1) diet and (2) exercise—and each part was covered by eight to nine questions. The purpose was to elaborate how this intervention study affected diet and routines related to exercise, motivations, and perceived barriers towards establishing new routines related to diet and exercise, reasons for maintaining newly established routines, and how diet and exercise affected their body. The interviews were transcribed using NVivo version 11.2.1 (QSR International, Doncaster, Australia). Nodes used to identify motivators and barriers for changes in diet and motivators and barriers for changes in physical activity were health, weight, diet, time constraints, social aspects, knowledge, and physical activity. Anonymity was emphasized, and participants could withdraw from the study at any time.

## 3. Results

### 3.1. Quantitative Results

In total, 98 participants completed the questionnaire after inclusion in the intervention: 51 (35 women) in TG (average weight 92.37 kg (87.34 to 97.4)) and 47 (30 women) in NTG (average weight 94.06 kg (89.12 to 99.00)). After 33 weeks (w), 71 participants completed the questionnaire, including 34 (23 women) in TG and 37 (23 women) in NTG; and 1 year after (1YA), 58 participants, 26 (17 women) in TG and 32 (20 women) in NTG, completed the questionnaire.

#### 3.1.1. Nutritional Guidance

Thirty-four participants (n = 58 respondents) reported having changed their diet due to the intervention study (72% in NTG and 42% in TG). Among those who did not change their diet, nine participants (6% in NTG and 27% in TG) reported that their diet was already in accordance with, or close to, the Norwegian Directorate of Health recommendations. Despite a healthy diet, the most common reason reported for their overweight or obesity was the amount of food prepared, meal size, and lack of willpower. Table 1 shows changes in consumption of healthy and unhealthy food as from entering the intervention and until 1YA follow-up.

All participants steadily increased the consumption of healthy food and decreased the consumption of unhealthy food during the intervention period. Pairwise comparisons indicate a statistically significant increase between baseline and 1YA for consumption of fruit (*p* = 0.016) and vegetables (0.004), and a decreased consumption of refined grains (*p =* 0.007), SSB (*p* < 0.01), junk food (*p* < 0.01), snacks (*p* < 0.01), and sweets (*p =* 0.001 (time) and 0.014 (group)). In addition, the consumption of sweet pastries significantly decreased from baseline and until 33 w (*p =* 0.01) and from 33 w until 1YA (*p =* 0.01). A significant increase in the consumption of legumes was seen during the first 33 w (*p =* 0.001). However, interaction effects of group x time were found for refined grains (*p =* 0.031), vegetables (0.019), legumes (*p* < 0.01), and sweets (*p =* 0.001) between baseline and 1YA. For refined grains, a significant interaction effect was also detected between 33 w and 1 YA (*p =* 0.009), while interaction effects for sweet pastries were detected for the first 33 w (*p =* 0.014). Significant differences between TG and NTG related to frequency of consumption were found for vegetables (*p =* 0.026) and legumes (*p* < 0.001) after 33 w. Additionally, from 33 w and until 1YA follow-up, results show a significant difference between groups related to consumption of refined grains (*p =* 0.016) and vegetables (*p =* 0.01).

At 1YA follow-up, participants were asked which of the 12 dietary guidelines they found most challenging. Overall, 20 persons in NTG (n = 31) and 13 in TG (n = 26) found guideline number two most challenging: to maintain a good balance between the amount of energy you obtain through food and drink and the amount of energy you expend through physical activity. The change that was easiest to implement was to use low-fat dairy products (stated by 24 in NTG (n = 31) and 22 in TG (n = 26)).

#### 3.1.2. Physical Health

All participants were asked about the three most important reasons for joining the project, and almost without exception, all participants reported the same goals: better shape, reduced weight, and to establish training routines and the joy of exercise. Answers were almost the same, disregarding BMI and gender. The most common factors mentioned by women were better health, physical shape, and weight. The most common factors mentioned by men were physical shape, weight, and diet. Participants graded their physical health when entering the intervention and 1YA. Independent of weight loss and group, participants reported that they were in better physical shape 1YA (1.81 ± 0.128 (NTG) and 2.06 ± 0.137 (TG), where value 1 indicates much better and value 2 indicates slightly better shape). Although not significant, this was a motivational factor if results from the anthropometric measurements were disappointing: “Focusing more on getting into shape, not just losing weight” (TG).

### 3.2. Qualitative Results

#### 3.2.1. Motivators for Changes in Diet and Physical Activity

According to the questionnaire and the interviews, the most important motivator for changing diet was related to health. They felt it was easier to concentrate, they managed to complete daily tasks and they generally felt better. “The slow carbs affect how your brain works” (TG). When eating healthily, it was easier to complete workouts, and participants experienced a feeling of wellness. “You feel it gets easier to do new things” (NTG).

Newly established routines consisted of consuming less fast food and preparing food with healthy ingredients. “I try to limit the amount of sweets during everyday life” (NTG). On the other hand, the most important reason for not losing weight was “snacking”, which was reported by eight participants.

The TG participants were asked not to change their diet during the intervention period, but some of them admitted that they did anyway: “Most of us know it’s better for us to have a salad than a burger, but it all comes down to making that choice. This choice was easier when I’d exercised, resulting in a continuous change in diet”. Others stated: “I’ve seen that there must be a balance between being physically active and having a healthy diet”; “When I’ve spent so much time on training, it seems foolish to return home to the sofa with a bag of crisps and a coke”.

On responding to the questionnaire, participants stated the five most important aspects related to exercise to be the following: (1) I can take responsibility for my own health, (2) I believe that physical activity is good for my health, (3) exercise is important in many aspects of life, (4) it is important to live as healthily as possible, and (5) I wish to be a role model for my children.

When analyzing factors of importance for the motivation to change an already established lifestyle, answers from men and women mostly coincided. Both mentioned improved physical health as the most important reason for joining the intervention. The ability to maintain or lose weight was also mentioned. Men also included diet, in addition to such words as “body” and “knowledge”, which were not mentioned by women.

Another focus was the importance of not giving up if you break good routines occasionally: “This project has taught me something, being able to only go back to point zero. Instead of giving up and thinking I cracked” (TG). In one of the interviews, one of the informants reported it was motivating that everyone in the group had been in bad shape and had started training at the same level: “What helped most then was that there were many others with the same type of form, or lack of form” (TG). In addition, maintaining physical fitness and new habits were important: “Now that you’ve got into good shape, it’s worth trying to keep it up. Don’t fall back on the couch again” (NTG). Being a group was also important, especially until they had established training routines of their own: “For me, the first three months had a great impact” (TG). Other important motivational factors were the observation that many of the participants gradually got into better shape, together with the presence of a professional, knowledgeable trainer at all joint exercises: “I saw that many were in better shape, because I eventually started talking to people”. During two of the interviews, it was also stated that seeing continuous progress on biometric measurements and fitness tests was important for their motivation. 

Overall, 16 participants in NTG (n = 31) and 11 in TG (n = 26) found it difficult to follow the advice related to physical activity. Among those who quit training after the intervention period, motivation and willpower were important factors in prioritizing and planning how to start up training again.

#### 3.2.2. Perceived Barriers That Overweight and Obese People Encounter When Starting to Exercise and/or Change Diet

Even though supportive families and friends were important factors in helping participants to manage to change their diet and eat more healthily, family could also be a limiting factor. Participants with children living at home were afraid that exaggerated focus on diet could increase children’s risk of developing eating disorders. “Afraid of eating disorders”, “must eat ordinary food” (NTG).

All four interviewees confirmed the results from the survey, stating that changing habits was an important challenge in managing to eat healthily. In everyday life, this challenge was mentioned especially together with limiting portion sizes and challenges when buying food, but also in relation to a busy life, business trips, and holidays with traditions involving the preparation and consumption of high-calorie food and sweets. On many occasions the type of food was not a problem, but rather the amount of food prepared and consumed. “Like I said now at Christmas. I love to bake and try new recipes. Confectionery and everything. And I must always taste it (laughing), right?” (NTG). They had the idea that healthy food required longer preparation time and that daily time constraints gave less time to prepare food, which in turn often led to unhealthy fast food. Planning what to buy and what to eat was important.

Results from the questionnaire showed that barriers related to physical activity were time, motivation, and self-discipline. One of the interviewees said that holidays present challenges to maintaining the volume of exercise. However, it might also be an opportunity to get to spend more time on exercise: “That you actually have better time to exercise. You do not have that extra hurdle when you get back home” (NTG). The time conflict between work and exercise at 4.30 p.m. was by most participants stated as the main reason for not attending this joint exercise 1YA, but when they did attend, being together gave extra energy: “I feel that I push myself a little bit extra when I exercise with others” (NTG).

## 4. Discussion

Both TG and NTG reported better shape 1YA than when joining the project and had a diet consisting of healthier ingredients and less unhealthy food. Despite being told not to change their diet, results show that the TG participants gradually changed to a healthier diet during the intervention period, by increasing their consumption of fruit and vegetables and reducing their consumption of SSB, junk food, refined grains, snacks, sweets, and sweet pastries. Interviews with TG participants revealed that it became natural to establish a healthy diet after spending time on exercise. An increased intake of fruit and vegetables is also associated with weight loss [3,33] and with amount of physical activity [34]. Results indicate that healthier eating habits develop because of exercise. This might be one reason for the lack of statistical significance between the groups after the intervention period, for other ingredients than legumes and vegetables, despite nutritional guidance only being given to the NTG participants. From an ethical point of view, it is difficult to deny participants to make this type of change, although the TG participants were strongly encouraged to not make any dietary changes during the intervention. Results show that 42% of the TG participants changed their diet during the project period and 27% reported to already be eating according to dietary advice. Among NTG participants, 72% changed their diet, but only 6% reported to already be eating according to the advice. After 33 w of nutritional guidance, answers from the FFQ show a significantly increased consumption of vegetables (*p =* 0.006) and legumes (*p =* 0.034), but this levelled out after ending their dietary courses, and 1YA there were no differences between the groups.

Combined interventions with diet and exercise are more effective than diet or exercise separately [2,3,4,35]. Results from this intervention show that participants will most probably change their diet as a natural consequence of starting to exercise, and that those who already exercise probably have a healthier diet, disregarding weight. Anderson, Konz, Frederich, and Wood [36] have also shown that those who exercised more were better able to maintain their weight loss than those who did not. Most of the participants still exercised regularly 1YA, implying that it might be easier to achieve a healthier diet. This was also supported by the interviews, where participants in this project reported better physical shape compared to baseline, results that were also documented by running time and weight measurements [26,27,28].

Both nutritional guidance and physical activity influenced diet. Mentally, both exercise and changing eating habits gave participants a better mood and a positive energy surplus. Wadsworth and Pendergast [37] reported a negative relationship between obesity and life satisfaction and a better mood might also be an important factor related to adherence [38]. However, not all participants were mentally ready to change their diet during the project period, but pictured themselves with a gradually healthier everyday life, which illustrates that lifestyle changes require follow-up over a long period of time. The adherence rate in this project was 59%, which correlates well with a reported average adherence rate of 60.5% in weight-loss interventions [19]. Changing habits is difficult, but research has shown that this might be easier if participants are offered supervised attendance, with social support and focus on dietary modification, instead of just exercise [19,39]. Both groups received supervised attendance and established a social network during exercise. Participants mentioned the importance of being part of a group until routines were established. Lemstra, Bird, Nwankwo, Rogers, and Moraros [40] also showed that offering social support improved the adherence rate by 29%. The NTG participants gained a closer relationship with the researchers and other co-participants during practical cooking sessions, and this might be one reason why the NTG participants had a higher adherence rate (68%) than the TG participants (50%). Although the NTG participants spend more hours together with the researchers, the TG participants also gained a close relationship with other participants and the researchers during the two weekly training sessions. In addition, participants could easily get in touch with each other through a closed Facebook group when they were in need for a training buddy. Interval training programs were also published in this group. A lower adherence rate in the TG group could also be caused by the dissatisfaction of not being able to change their diet. Most of the applicants wanted to belong to the NTG. Some reported injuries as their reason for dropout, although most dropouts failed to show up for measurements and were independent of BMI. Having family was a support, but also an obstacle on the way to a healthier diet, as was also stated by Austin, Smith, Gianini, and Campos-Melady [41]. It was difficult when family members had different nutritional needs or food preferences. Both groups reported work and a busy everyday life as the main causes for not participating in joint training and an important reason for dropout. This also coincides with causes of dropout reported by Meffert and Gerdes [10].

Many factors have been identified as contributing to obesity, including the food environment, especially the access, availability, and affordability of healthy foods in grocery stores and supermarkets [42]. Easy access to healthy food was also mentioned as a prerequisite for eating healthily in this project. Hall and Kahan [43] refer to the obesogenic environment and highlight the industrialization of the food system involving ultra-processed food as a causative factor for weight gain. They also mention the importance of home-prepared meals. Learning and practicing basic cooking techniques and methods might make intervention participants more eager to prepare their own food and make it easier to establish a healthy lifestyle. In this intervention, participants became familiar with different healthy ingredients and cooking techniques to establish a varied diet, and participants were also eager to prepare these meals at home. In the ranking of dietary advice, participants found it easy to replace hard margarine and butter with oil/soft margarine. The idea that oils are considered healthier than butter (related to lifestyle diseases) may have led to greater consumption, even though oils are more energy-rich than butter. Nutrient knowledge is often poor in obese [44]. Knowledge about food is important for choosing healthy ingredients and composing meals according to recommendations, and increased nutritional knowledge is associated with healthier eating habits and weight maintenance after interventions [44,45]. After a nutritional educational intervention for eight months, López-Hernández, Martínez-Arnau, Pérez-Ros, Drehmer, and Pablos [45] showed a significant reduction in the consumption of sweets, soft drinks, high-fat products, and processed meats, and an increase in the intake of lean meat and poultry, together with a 3% decrease in body weight. In this project, “knowledge” was stated to be particularly important for male participants. According to Taylor et al. [46], engaging males in nutrition programs may require different approaches than for women and require food-based guidelines, and the information presented must be clear, concise, and provided in an engaging and enjoyable way. Male participants also mentioned “body” as important, probably of importance as an external factor, but also because they had been made aware of their visceral fat result [26,28]. Interviewed participants started to read cookery books and used less processed food in their everyday diet. They prepared most of their food from healthy ingredients, consisting of more vegetables and wholegrain products, while ingredients high in sugar were mostly consumed during weekends. However, the energy density of healthy food is often underestimated, and portion estimates are larger than recommendations [47]. One of our participants confirmed this: ”Used to love nuts. Found out that one bag of nuts gave me 1200 Kcal…so. We think nuts are so healthy” (TG). Additionally, McCrickerd et al. [48] have reported a connection between portion size and high dietary energy density. Most participants with less favorable weight loss were aware of the reasons for this, such as snacking, stress, injuries, too much alcohol, large meals at night (after doing well all day), large portions in general, and not enough exercise, with snacking being the most important reason. Several participants had a healthy diet, as defined by the Food Pyramid and the Norwegian Directorate of Health. However, they were unaware of the fact that their weight could increase due to large portions, even if they ate healthily. It is important knowledge that healthy food may also be high in calories. Healthy habits were also often forgotten at social events and parties. It was a challenge to avoid unhealthy food, and planning purchases were reported as a crucial factor for eating healthy food/fewer calories or smaller portions. Pedersen et al. [49] have also shown the importance of habitual routines for long-term weight loss maintenance to ensure stronger self-control in the behaviors related to weight loss maintenance, such as buying and storing food and eating at social gatherings. This gives more flexibility, in contrast to short-term maintainers who mostly focus on avoidance [49]. Changing shopping habits in the form of a note on what food to buy, shopping for many days at a time and avoiding shopping when hungry made it easier to avoid purchasing unhealthy foods, besides the fact that they had to decide for themselves (internal decision) that they wanted to eat more healthily.

### 4.1. Motivators for Changes in Diet and Physical Activity

The most important motivators for changing diet were health benefits and to lose weight. A small weight loss is sufficient to reduce lifestyle diseases [50]. van Uffelen et al. [51] also reported the three most important factors for physical activity to be to prevent health problems, to feel good, and to lose weight. In addition to exercise, the consumption of healthy food and limiting amounts of sweets on weekdays were key contributors to maintaining weight loss in both groups in this study.

Regular anthropometric measurements helped the participants to be active and to eat healthier during and after the intervention. Practical cookery lessons were motivating as an arena for discussion of recipes and how to cope with everyday life, and the acceptance of obesity as a chronic disease that requires treatment across the lifespan [50].

Better physical shape was an important motivator for many of the participants. Results from the questionnaire and interviews showed surplus physical and mental reserves and that they had more positive energy in everyday life.

Exercise together with untrained, overweight people who were in as bad shape as oneself was motivating. Even though the intensity of the interval training was high, it emerged from the interviews that they did not need to set a new personal record at every training session. According to van Uffelen, Khan, and Burton [51] both men and women preferred free activities close to home, preferably activities that could be done alone, although women were less competitive and preferred less vigorous activities than men. After a few weeks of training, they already communicated a significant increase in physical fitness, and after 4–6 months, exercise routines were established. Together with mastering experiences and handling of relapses, this may have been a reason to continue to exercise 1YA.

Participants found it motivating that the project was knowledge based. The interviews revealed differences between NTG and TG participants in the use of terms related to diet. Not unexpectedly, NTG participants displayed more nuanced language and a better understanding of diet/food in the interviews than TG participants.

### 4.2. Perceived Barriers When Starting to Change Diet and/or Exercise

The participants wanted to learn more about training and nutrition, and they felt confused by conflicting advice about diet in the media. Based on results from the interviews, some of the participants seemed to consider the dietary advice given in this project to be a “special diet” and not a lifestyle. They felt they had to hide their “diet” from their children and cut all snacks and alcohol, indicating a lack of knowledge that these 12 Norwegian guidelines are recommended for everyone, independent of age and BMI. It is likely that this knowledge was an important motivator in maintaining both the activity level and a healthier diet.

Barriers might be a lack of internal motivation to be physically active or an unhealthy body image. The questionnaire responses indicate that some participants were still at stage two in the Stages of Change Model [7,8] after the intervention. They acknowledged the problem and knew what changes they ought to make but were not in bad enough shape/health for these changes to be crucial. They would act, however, if their weight continued to increase. Some participants had the willpower to change and were at stage four. Willpower is a crucial factor to be able to establish healthy eating habits. According to Pinho et al. [52], lack of willpower, time constraints, and taste preferences were the factors most consistently associated with low consumption of healthy food and a high consumption of unhealthy food. Lack of willpower has also been connected to low consumption of fruit, while low consumption of vegetables can be related to a busy everyday life [52]. In the study by Mc Morrow et al. [53], willpower was also reported to be one of the most important barriers to eating recommended amounts of fruit and vegetables among women, together with low cooking skills, not liking the taste of healthy food, and preparation time [53]. Men did not like the taste of fruit and vegetables or found them boring [53]. Kwasnicka, Dombrowski, White, and Sniehotta [54] reflected on challenging environments (hotels, energy-dense snacks at hotels) in their study, and this was also the case for some participants in this study, who were continuously offered free food and drinks during work-related travel, together with frequent cake servings at work. They felt under pressure to eat, while also being supervised by people giving advice regarding their eating habits. Changing behaviors, breaking old dietary habits, and making new ones resembled a struggle between the individual’s “good” and “bad” sides and seemed to cause conflict with identity or self-awareness, as also described by Green, Larkin, and Sullivan [55].

Both weight re-gainers and maintainers mention weight management as a constant battle leading to cognitive fatigue, which in turn leads to a negative spiral and poor coping strategies, and eventually relapse [56]. The participants in the current study reported exercise to be a positive coping strategy for reducing this type of battle. To lose weight, it might be wise to start to exercise, and as routines are established, diet changes will follow. One TG participant said: “Yes, really it hurts to isolate only the diet, but … But … it’s important to have a physical surplus for exercise. So, I really think it’s mostly the exercise that makes me feel mentally good.” Mikkelsen et al. [57] also showed the importance of exercise in relation to positive mental well-being.

A few participants had a diet in accordance with the nutritional recommendations and knowledge about diet when joining the project, but needed to reduce their meal size. It was a struggle to achieve a good balance between the amount of energy from food and drink and the amount of energy expended through physical activity. Compensatory beliefs may impede pursuit of the goal. The impact of goal-consistent exercise behavior seems to be greater than the impact of goal-inconsistent eating behavior [58]. As an example of compensatory beliefs, some participants consumed chocolate or energy bars after exercise. Changes in appetite seem to be more important to explain the plateau in weight loss than slowing metabolism. They embellished their exercise and downplayed their eating. Increased energy expenditure due to exercise also leads to a greater feeling of hunger, followed by a concomitant increase in calorie intake that may prevent further weight loss [59]. Polidori, Sanghvi, Seeley, and Hall [6] estimated a calorie expenditure of 20–30 kcal/day for each kilogram of lost weight, whereas the appetite increases about 100 kcal/day, giving a calorie surplus.

Regular exercise was challenged by time pressure: “But it is to make time for it”, “Not enough time”. This was also one of the factors underlying weight regain identified in the reviews of Ulen, Huizinga, Beech, and Elasy [39] and Oh et al. [60]. Hoare et al. [61] also identified lack of time as the most frequently reported barrier to physical activity among inactive adults. High-, low- and moderate-intensity training seem to have the same effect on body weight, but high-intensity training, requiring about 40% less time commitment, and running seem to be more effective on body composition measures compared with other activities [62]. Interval training is highlighted as positive by the informants in this study and may therefore be recommended for lifestyle changes.

Adhering to a healthy diet and regular exercise is required for weight loss both initially and in the long term. It is also of interest to see if overweight and obese respond differently to an intervention study regarding dietary advice or physical activity, as shown by Herrera-Espiñeira et al. [63]. Adherence to the program is important to losing weight and reasons for adherence, or the lack of it, should be studied further. The link between knowledge about nutrition and degree of overweight is also interesting and ought to be studied in more detail, although results from this intervention also indicate that regular exercise will automatically lead to a healthier diet.

## 5. Conclusions

Although the NTG ate significantly more vegetables and legumes than the TG after the intervention, no significant differences in dietary habits were found between the groups at one year follow-up. As training became routine, all participants started to eat more healthily, independent of intervention group, which indicates that exercise routines have an impact on diet. General health was an important motivator for dietary change and exercise. Planning purchases and regular exercise were important factors to achieve and maintain lifestyle changes. Reduced weight and other physical and psychological aspects of exercise were important to make dietary changes and continue exercise. Barriers to changing diet were related to work, family, meal size, and the internal decision to change habits. Barriers to exercise were holidays and time constraints. Establishing training routines will naturally affect diet in a positive way, potentially on the same level as dietary guidance. For healthy overweight and obese, it is important to focus on lifestyle changes according to national dietary recommendations. Interventions based on individuals’ motives for physical activity and dietary change could improve effectiveness. Future research should examine this natural change in diet due to supervised training and the internal motives for changes in diet and physical activity.

### Strengths and Limitations

One limitation of the current study is the relatively small number of participants. However, limited resources in the active part of the intervention study made it impossible to enroll more participants, and this combined with the high secession rate in TG meant that the number of completers was low. Participants were recruited through a local newspaper with a circulation of about 65,000 newspapers at the time of enrollment. The ad was open, which meant that there was no need for a subscription to read it, but it may have affected the representativeness of the group. The study is dependent on dietary recall. Despite these limitations, this exploratory, randomized trial helps to identify important associations between weight and motivation for physical activity and an improved diet. The strength of this study is the duration of two years, with three measurements during this period.

## Figures and Tables

**Table 1:** Baseline characteristics and average changes in frequency of consumption, expressed as servings per week.

Variable	Baseline Means ± SD		Post Intervention Change (33 Weeks) (5th 95th Percentile)		1YA Follow Up (5th 95th Percentile)	
	NTG	TG	NTG	TG	NTG	TG
**Fruit**	6.4 ± 3.94	5.25 ± 4.0	0.86 (−0.41 to 2.14)	0.35 (−1.1 to 1.78)	1.6 (−0.04 to 3.24) †	0.70 (−0.9 to 2.3) †
**Vegetables**	5.59 ± 3.58	5.13 ± 3.77	2.46 (1.16 to 3.76) *	0.30 (−1.13 to 1.73) *	1.2 (−0.39 to 2.79) †,¶	1.76 (0.47 to 3.06) †,¶
**Wholegrains**	6.09 ± 3.33	6.8 ± 3.74	0.7 (−0.66 to 2.06)	−1.15 (−2.52 to 0.23)	0.39 (−0.85 −1.62)	0.27 (−1.23 to 1.78)
**Fish**	2.06 ± 1.87	2.64 ± 2.65	0.19 (−0.57 to 0.93)	−0.42 (−1.13 to 0.28)	0.59 (−0.47 to 1.65)	−0.28 (−1.22 to 0.66)
**Legumes**	0.7 ± 1.13	0.65 ± 0.94	0.78 (0.47 to 1.09) *,‡	−0.02 (−0.31 to 0.27) *,‡	0.45 (0.15 to 0.75)	0.09 (−0.51 to 0.68)
**SSB ¹**	2.22 ± 2.83	2.42 ± 3.52	−1.49 (−2.34 to −0.65)	−0.55 (−1.5 to 0.40)	−1.37 (−2.14 to −0.59) †	−0.71 (−1.59 to 0.18) †
**Junkfood**	1.33 ± 1.35	1.21 ± 1.19	−0.53 (−0.91 to −0.15)	−0.46 (−0.83 to −0.09)	−0.43 (−0.82 to −0.03) †	−0.57 (−0.9 to −0.24) †
**Refined grains**	1.59 ± 1.88	1.40 ± 1.95	−0.55 (−0.99 to −0.1)	−0.19 (−0.59 to 0.22)	−0.76 (−1.18 to −0.34) *,†,¶	−0.18 (−0.64 to 0.28) †,¶
**Salty snacks**	1.84 ± 1.31	1.97 ± 2.07	−0.71 (−1.15 to −0.27)	−0.5 (−1.1 to 0.08)	−0.70 (−1.07 to −0.33) †	−0.43 (−0.79 to −0.07) †
**Sweets**	3.14 ± 2.81	1.91 ± 1.6	−1.65 (−2.6 to −0.7)	−0.25 (−0.71 to 0.22)	−2.05 (−3.1 to −1.0) †	0.02 (−0.52 to 0.55) †
**Sweet pastries**	1.57 ± 1.89	1.06 ± 1.1	−1.19 (−1.87 to −0.51) ‡	−0.23 (−0.65 to 0.19) ‡	−1.29 (−2.06 to −0.53) ∆	−0.36 (−0.73 to 0.00) *,∆

^¹^ Sugar Sweetened Beverages. * Statistically significant difference between groups (*p* < 0.05). ¶ Statistically significant difference between groups from 33 w until 1YA (*p* < 0.05). † Pairwise (TG + NTG) statistically significant difference from baseline until 1YA (*p* < 0.05). ‡ Pairwise (TG + NTG) statistically significant difference from baseline until 33w (*p* < 0.05). ∆ Pairwise (TG + NTG) statistically significant difference from 33w until 1YA (*p* < 0.05).

## Data Availability

The data presented in this study are available on request from the corresponding author.
